# An evergrowing sweet cherry for research and breeding

**DOI:** 10.3389/fpls.2025.1677862

**Published:** 2025-11-26

**Authors:** Afif Hedhly, Nerea Martínez-Romera, Ana Pilar Gracia, Juan Marin, Arancha Arbeloa, Elena García, Ana Wünsch

**Affiliations:** 1 Department of Plant Sciences, Centro de Investigación y Tecnología Agroalimentaria de Aragón (CITA), Zaragoza, Spain; 2 Instituto Agroalimentario de Aragón-IA2 (CITA-Universidad de Zaragoza), Zaragoza, Spain; 3 Department of Pomology, Estación Experimental de Aula Dei (EEAD-CSIC), Zaragoza, Spain; 4 Fundación Agencia Aragonesa para la Investigación y el Desarrollo (ARAID), Zaragoza, Spain

**Keywords:** *Prunus avium*, evergrowing, evergreen, seasonal growth, dormancy, dormancy associated MADS-box genes, *DAMs*, inbreeding

## Abstract

Evergrowing phenotypes in deciduous trees have only been described in three unrelated species: peach, hazelnut and pomegranate. These genotypes are a useful tool for forward genetics approaches aimed at understanding the processes that regulate seasonal growth and dormancy. Research in evergrowing peach lead to the identification of the DORMANCY ASSOCIATED MADS BOX transcription factors (*DAMs*) as regulators of dormancy in stone and pome fruits. In this work we present the breeding and preliminary characterization of an evergrowing (evg) sweet cherry genotype. This individual was obtained from *in vitro* embryo rescue of self-fertilization seeds, of a local self-compatible landrace. Unlike wild type sweet cherry, evg sweet cherry does not set buds in autumn and continues to grow throughout the winter. In contrast to evergrowing peach, no major structural deletion was observed in the tandemly arranged *PavDAMs* genes. However, specific expression profiles of these genes were observed in the evg sweet cherry. The specific polymorphisms previously observed in the *PavDAMs* of the parental cultivar, and the high level of inbreeding depression resulting from self-fertilization, suggest that the expression of homozygous recessive alleles might be the cause of the evergrowing phenotype. Ongoing work to fully characterize the underlying molecular mechanism regulating evg sweet cherry phenotype is discussed, highlighting its importance and utility as a research and breeding tool.

## 1 Introduction

Sweet cherry (*Prunus avium* L.) is a temperate fruit tree species of high economic interest. It belongs to the *Prunus* genus together with other important temperate fruit species like peach (*P. persica*), plum (*P. domestica*), apricot (*P. armeniaca*) and almond (*P. dulcis*); and to the Rosaceae family, which also includes economically important pome fruit trees such as apple (*Malus domestica*) and pear (*Pyrus communis*). These species undergo a dormancy period during the winter, when growth stops and resumes in spring, once the cold and warmth needs are satisfied. Environmental cues, such as temperature and photoperiod, are sensed to adjust growth and phenophases (Singh et al., 2017). In temperate fruit trees, optimum production relies on the adaptation of the plant material to environmental conditions. In particular, winter chill requirements and bloom date are phenological traits on which the choice of cultivars is based to ensure adequate flowering and fruit development in a given region. Current and future climate scenarios, with mild winter temperatures, are about to alter this finely tuned plant-environment interaction (Kovaleski, 2024). Dormancy and blooming time are therefore becoming important breeding traits in temperate Rosaceous fruit trees, and great efforts are being made to understand their underlying molecular mechanisms.

Recent advances in the understanding of flowering time control using model plant species have led to the identification of several pathways that cross talk through a few major transcription factors called floral integrators. These include several MIKC^c^-type transcription factors such as *FLOWERING LOCUS C (FLC)*, and *SHORT VEGETATIVE PHASE (SVP)*, that repress the expression of the often referred to as florigen *FLOWERING LOCUS T (FT)*, and *SUPPRESSOR OF OVEREXPRESSION OF CO 1 (SOC1)*, resulting in the inhibition of the transition to flowering (reviewed in Teotia and Tang, 2015 and Xu and Chong, 2018; Wang et al., 2025). Interestingly, some of these flowering pathways also regulate seasonal growth in woody plant species. Homologous or orthologous genes to floral integrators have been shown to mediate autumn growth cessation, winter dormancy and spring sprouting in model forest tree species (e.g. Böhlenius et al., 2006; Singh et al., 2018, 2019, André et al., 2022; Ding et al., 2024). Environmental cue sensing that underly growth cessation and dormancy has been shown to be different in some Rosaceae temperate fruit tree species (Singh et al., 2017; Zhang et al., 2025), warranting similar strategies in these species.

The progress in understanding flowering time and seasonal growth regulation in temperate fruit trees is lagging, mainly because of the limited number of mutants and the difficulty of regenerating knock-out and overexpression transformants, especially in *Prunus* species (see Vergara et al., 2021 for recent progress). However, important progress has been made through the characterization of natural spontaneous evergrowing mutants. In peach, the identification, characterization and mapping of an evergrowing genotype (Rodriguez-A et al., 1994; Wang et al., 2002) revolutionized the understanding of dormancy in the Amygdaloideae subfamily by uncovering a prominent role of a tandem set of MIKC^c^-type transcription factors within the SVP/AGL24 clade, named *DAM* genes. Deletion of 4 of the 6 *DAM* genes in the evergrowing peach leads to the non-dormant evergrowing phenotype (Bielenberg et al., 2004; 2008). However, pathway(s) involving *DAM* genes, and function are not yet fully understood. In subtropical pomegranate, the recent characterization of an evergrowing phenotype revealed that the phenotype is also caused by a mutation in a MIKC-type MADS box gene, highly similar to the Arabidopsis *FLC*-clade member *Agamous-like 27* (*AGL27*), also known as *Flowering Locus M 1 (FLM1*) or *MADS Affecting Flowering 1* (*MAF1*), with a unique glutamine repeat motif (PolyQ) in its N-terminal region (Harel-Beja et al., 2022). While these results highlight the prominent role played by MIKC^c^-type transcription factors of the *FLC* and *SVP/AGL24* clades in the perception of environmental cues that allow trees to adjust their growth and flowering, more mutants and mutagenesis approaches are needed in fruit tree species, to resolve the underlying molecular mechanisms.

In 2008-2009, the development of a self-fertilization population (F2) from the self-compatible local sweet cherry cultivar (‘Cristobalina’), for genetic mapping and genetic analyses (Calle et al., 2018), led to the unexpected obtention of evergrowing plants (A. Wünsch, unpublished results). These plants were planted in the field but did not survive in field growing conditions. In 2021, as part of a sweet cherry breeding project aimed at generating new phenotypic diversity with low chilling requirements, self-fertilization seeds of the same cultivar were newly germinated to obtain the same evergrowing phenotype. One individual exhibiting an evergrowing phenotype was obtained. In this work, we present a preliminary morphological and molecular characterization of this valuable plant material and discuss its possible causes and biotechnological usefulness. As a perspective, we propose that inbreeding in this highly heterozygous species may be an important source for generating unique genotypes for forward genetics approaches.

## 2 Materials and methods

### 2.1 Obtention and phenotypic characterization of sweet cherry evg

Sweet cherry cv ‘Cristobalina’ (wt-p; **Table 1**) trees located in CITA de Aragón, sweet cherry cultivar collection in Zaragoza (Spain) were used in this work. Fully mature fruits (n=173) from the open pollination of these trees were collected in May 2021 (66 days after full bloom, regular maturity period of this cultivar; Calle and Wünsch, 2020). Seeds were extracted and disinfected by washing with Sodium hypochlorite (10 g/l active chlorine) for 15 min and rinsed (3 times) with sterile distilled water. Integuments were removed aseptically, and well developed embryos (n=72; 42% of total fruits collected) were transferred to test tubes with C2D culture medium (Chée and Pool, 1987; Arbeloa et al., 2009). Test tubes were placed at 5.5 °C in the dark for 6 weeks, and then at 23 °C under cool-white, fluorescent light (50 μmol.m^-2^.s^-1^; 16h light/8h dark). Shoot tips from germinated embryos (n=24, 33% of cultured embryos, 16% of total fruits collected) were micropropagated *ex vitro* (Andreu and Marín, 2005) in DKW culture medium (Driver and Kuniyuki, 1984) to allow shoot multiplication. Shoots were rooted *ex vitro* (Castillo and Marín, 1994) after a quick dip (30 s) in K-IBA (1 mM) and grown with a peat–perlite substrate (1:1) under 100% RH in the greenhouse. Rooted shoots were acclimated (Marin, 2003) and transplanted into pots with the same substrate. Hence, several clonal plants were obtained of the different germinated embryos.

**Table 1:** Information on plant materials, dormancy phenotype and self-incompatibility genotype. *S*-locus and *MGST* genotypes analyzed to establish hybrid identity.

Cultivar/ individual	Name/ code	Relationship	Dormancy phenotype	*S*-locus genotype (H/h)	*MGST* genotype (H/h)
Cristobalina	wt-p	Parent	Wild Type	*S_3_S_6_* (H)	*wt/ins* (H)
Evergrowing cherry	evg	Self-pollination progeny	Evergrowing	*S_3_S_6_* (H)	*ins/ins* (h)
Sibling # 1	wt-s1	Self-pollination progeny	Wild Type	*S_3_S_3_*(h)	-/*ins*
Sibling # 4	wt-s4	Self-pollination progeny	Wild Type	*S_3_S_6_*(H)	*wt/ins* (H)
Sibling # 6	wt-s6	Self-pollination progeny	Wild Type	*S_3_S_3_*(h)	*ins/ins* (h)

‘wt’: wild type phenotype; ‘evg’: evergrowing phenotype; ‘*wt*’: wild type *MGST* allele; ‘*ins*’: mutated allele with insertion conferring self-compatibility; ‘h’: homozygous; H’: Heterozygous. -: data not available.

During the first year of growth, evg clonal plants and three randomly selected wild type full-siblings (wt-s1, -s4, -s6; **Table 1**) were monitored in a temperature-controlled greenhouse and in an outdoor shade-house. Preliminary phenotypic profiling consisted of a morphological characterization throughout the dormancy period monitoring leaf development, bud set, leaf fall, bud break and re-growth.

### 2.2 Preliminary genotypic characterization

Genomic DNA was extracted from leaf samples of the four genotypes (evg, wt-s1, -s4, -s6; individual potted plants as experimental unit), and *S*-locus analysis was carried out by PCR amplification of the *S*-locus genes *S-RNase* and *SFB*, using the primers PaConsI-F/PaConsIR2 and FBOX5´A/F-BOXintronR (Sonneveld et al., 2006; Vaughan et al., 2006) following the protocol described by Cachi and Wünsch (2014). Testing for the presence of insertion within the *MGST* gene conferring self-compatibility (SC) from ‘Cristobalina’, was carried out as described by Čmejlová et al. (2023) with modifications. Fragment analyses of all PCRs were carried out by capillary electrophoresis using SeqStudio (Thermo Fisher) genetic analyzer, size standard GeneScan 500-LIZ (Thermo Fisher), and analyzed and visualized using Genenious Prime^®^ 2025.0.3.

### 2.3 *PavDAMs* genes conservations and expression in evg

Genomic DNA of the evg sweet cherry, the parental genotype (wt-p), and two siblings (wt-4 and -6) were used to check the conservation of *PavDAM* genes by PCR analysis. PCR was carried out for each *PavDAM* gene using specific primers (**Supplementary Table 1**), at an annealing temperature of 59°C. PCR fragments were analyzed by gel electrophoresis. Reverse Transcription PCR (RT-PCR) was used to analyze the expression of the *DAM* genes in the same genotypes. Shoot apices or buds (depending on the growth stage of each individual at each sampling date) were sampled and immediately frozen in liquid nitrogen. Sampling was carried out at regular intervals during seasonal growth and dormancy (September to May). RNA was extracted using the ‘RNeasy Plant Mini Kit’ (Qiagen) following the manufacturer’s instructions. Reverse transcription was carried with using 2 µg of RNA using the ‘High-Capacity cDNA Reverse Transcription Kit’ (Applied Biosystems) following the manufacturer’s instructions. cDNA PCR was carried out for each *PavDAM* and a reference gene *Actin* using specific primers (**Supplementary Table 2**). PCR products were amplified with the following conditions: 94 °C for 4 min, 34 cycles of 94 °C for 45 s, 59°C (for Actin, *PavDAM-1,-4,-6*) or 57°C (for *PavDAM-2,-3,-5*) for 45 s, and 72 °C for 2 min, followed by a final extension at 72 °C for 7 min. All PCR fragments were separated on 1.7% (w/v) agarose gels and 1x TBE buffer run a 7 V/cm and stained with GelRed. The experimental units consisted of individual orchard trees (wt-p) or individual potted plants (evg, wt-4, and wt-6).

## 3 Results and discussion

### 3.1 An evergrowing (evg) cherry

**Figure 1:**
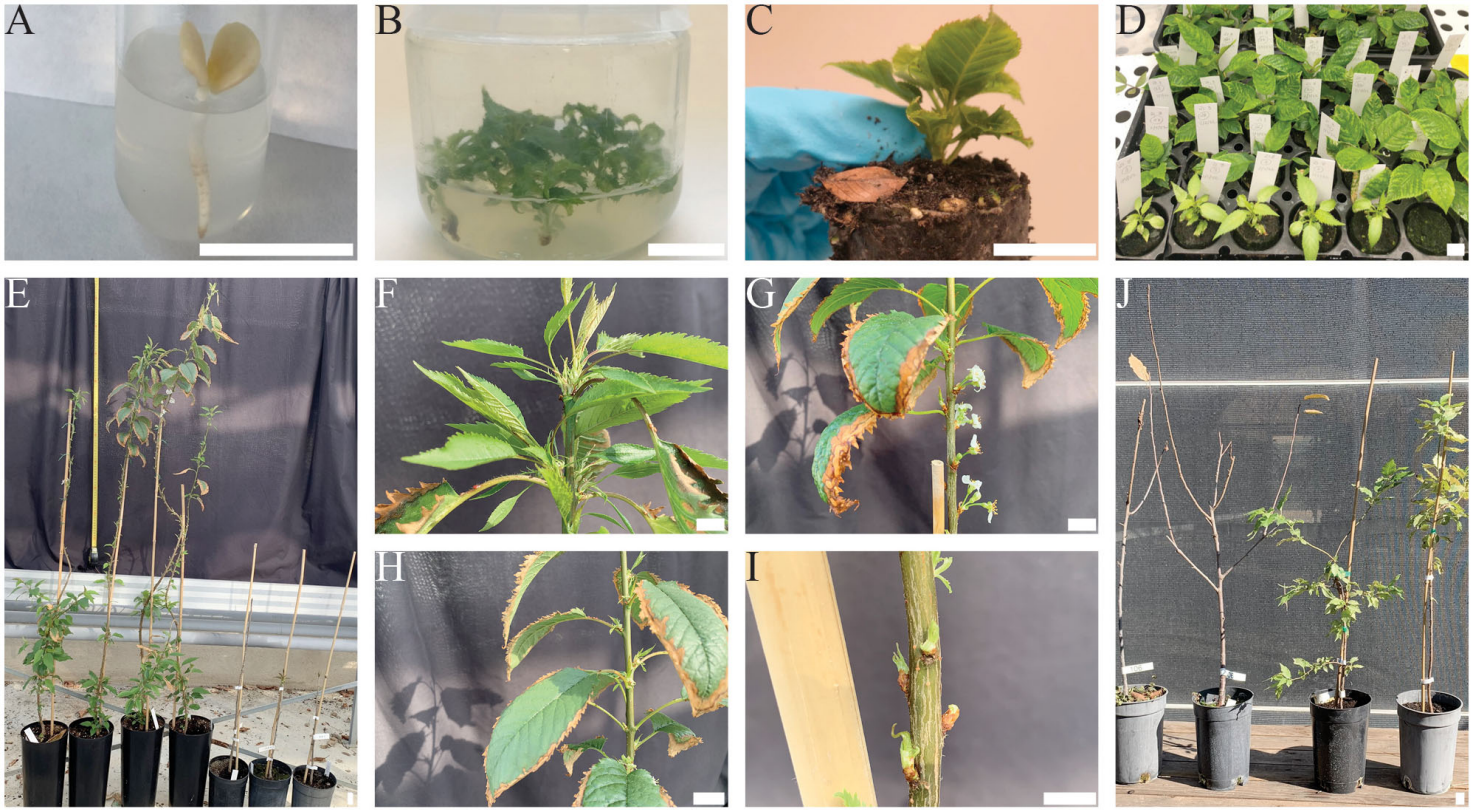
*In vitro* embryo culture and establishment of sweet cherry evg. **(A)** Germinated embryo. **(B)** Micropropagation of seedlings. **(C)** A rooted and acclimated seedling. **(D)** Acclimated seedlings in tray. **(E)** Seedlings in their first-year dormancy cycle. Notice the difference in height between wild type (right) and evg seedlings (left). **(F–I)** Close-ups of abundant leaf development **(F)**, flowering **(G)**, leaf margin desiccation before shedding **(H)**, and lateral vegetative buds **(I)** in evg plants. **(J)** Comparison of 2 wild type individuals that shed their leaves (left) at dormancy onset (October 2024), with two evg replica plants retaining their leaves (right). Bars = 2 cm.

Among germinated plants from embryo rescue of open pollinated ‘Cristobalina’ seed progenies (**Figures 1A–C**) an individual with a strikingly different phenotype was observed (**Figure 1D**). After acclimation, potting and transferring to greenhouse, while all normal-looking seedlings showed a typical cherry seedling phenotype, this offspring showed unusually continuous growth throughout the year and did not shed its leaves in autumn (**Figures 1E–J**). Left unpruned under warm greenhouse conditions, while wild type seedling reached an average height of 48 cm (SD 14), evg clones reached 160 cm (SD 36) cm at the at the end of growing season (**Figure 1E**). This phenotype showed profuse leaf and lateral shoots development (**Figure 1F**), and few flowers, developed from lateral shoots (**Figure 1G**), appeared at the end of its first growth cycle. Aged leaves develop a browning all around the leaf limb (**Figure 1G**) before falling. Under shade house conditions the following years, although the growth rate appeared similar between the wt and evg replica plants, the evg phenotype was maintained (**Figure 1J**) but flowers did not occur. This phenotype is similar to evergrowing individuals described in peach (Rodriguez-A et al., 1994).

‘Cristobalina’ is an exceptionally early flowering cultivar for which there is no flowering overlap with other cultivars in the collection and growing conditions. Thus, open pollinated seedlings are expected to originate from self-pollination. To confirm this assumption and confirm the origin of evg from self-pollination of ‘Cristobalina’, we used two complementary genetic analysis approaches. First, evg and its wild type siblings (wt-s1, -s4, and -s6) were analyzed with molecular markers to identify the self-incompatibility genotype at the *S*-locus. Since the wild type parental cultivar ‘Cristobalina’ (wt-p) *S*-genotype is *S_3_S_6_* (Wünsch and Hormaza, 2004), inbred seedlings are expected to be *S_3_S_3_*, *S_3_S_6_*, or *S_6_S_6_*(**Table 1**). The evg and all three wt seedlings had indeed the expected genotypes (**Table 1**, **Supplementary Figure 1**), with evg *S*-genotype being *S_3_S_6_*. Second, the self-compatibility of ‘Cristobalina’ is originated from a pollen part mutation unlinked to the *S*-locus (Wünsch and Hormaza, 2004, Cachi and Wünsch, 2011), namely an insertion in the *MGST* gene (Ono et al., 2018). ‘Cristobalina’ is heterozygote for the mutation, and only pollen carrying the mutation, either *S_3_* or *S_6_*, is capable of siring seeds. As a result, self-fertilization offspring should also carry the self-compatibility mutation. All full-siblings (wt-s1, -s4, and -s6), as well as evg were found to carry the mutated *MGST* allele, either in homozygosis or heterozygosis (**Table 1**, **Supplementary Figure 1**), further confirming the inbred origin of evg and wt-analyzed siblings.

### 3.2 Sweet cherry evg does not set buds or go dormant

**Figure 2:**
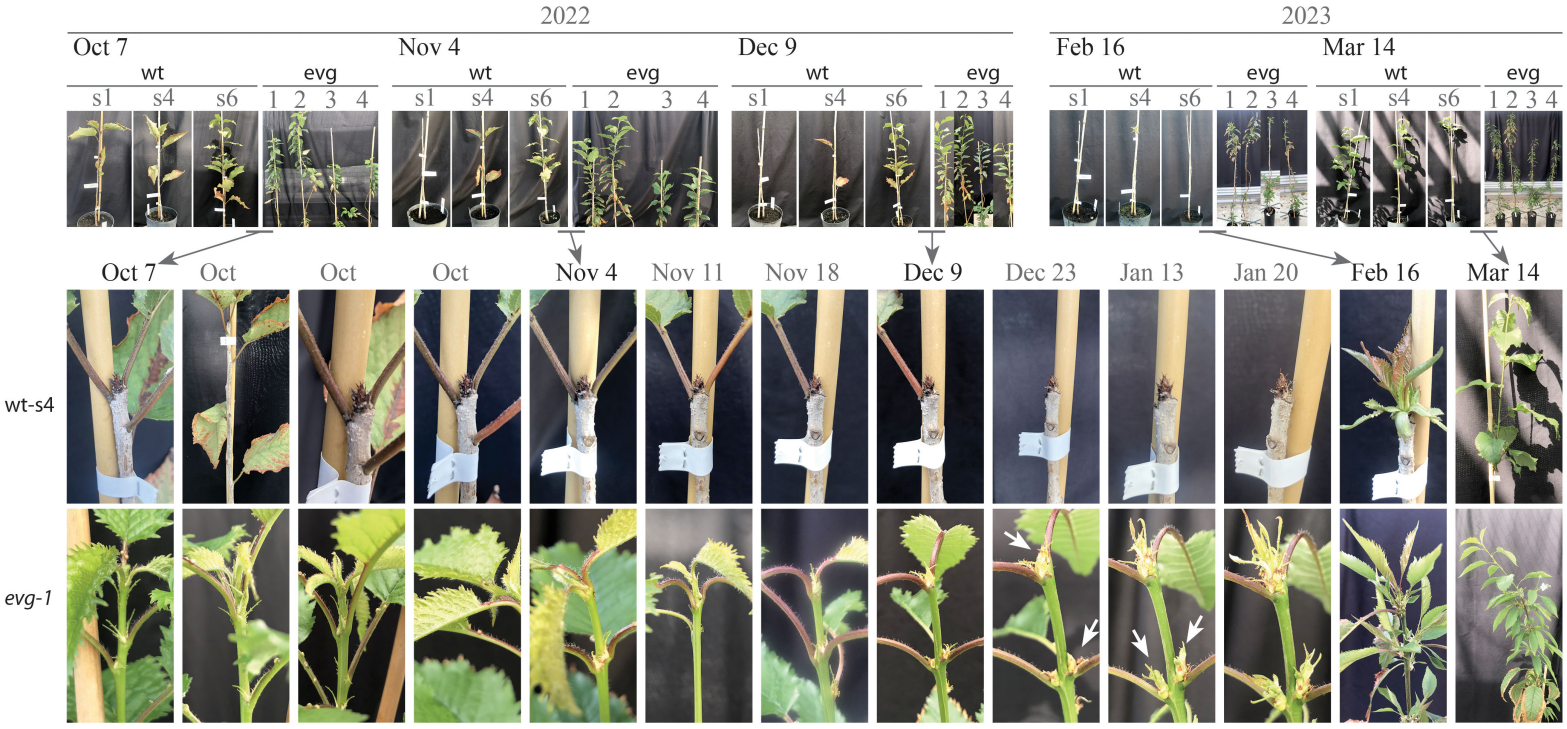
Sweet cherry evg does not set buds or go dormant. A developmental time series during the 2022–2023 dormancy cycle for 3 wild type individuals and 4 evg clonal plants. Whereas all wild-type individuals set terminal buds before the onset of the developmental time series and lost their leaves by the end of December, evg plants neither set terminal buds nor lost their leaves. In December, an apparent slowing of evg growth was observed (white arrows indicate buds not fully set), but it continued to grow throughout the dormancy period. .

To characterize in more detail the behavior of evg during dormancy, three wild type full-siblings and four evg replicas plants were weekly photographed paying special attention to the terminal shoot meristem and the first axillary buds below (**Figure 2**). At the onset of the developmental time series, on early October, while the three wild type individuals already had terminal buds and presented the typical brown leaf senescence spots that preceded their fall, the four evg plants were still growing actively and developing young leaves (**Figure 2**). Leaf fall dynamics varied among wt individuals, ranging from complete leaf fall in early November (wt-s1) to progressive leaf fall throughout most of the winter (wt-s6) (**Figure 2**). In evg plants, as in wt individuals, only the old leaves -away from the shoot tip- are shed. Although evg plants did not fully set the typical closed, compact, brown terminal buds, they appeared to slow their growth during the December to January period. However, the terminal and axillary shoots that appeared to have initiated the transition to closed bud structure, never matured into closed buds before the resumption of growth with increasing temperature. In fact, with increasing temperatures in February, evg individuals resumed growth earlier and faster than wt individuals.

### 3.3 *PavDAM* genes are distinctly and differentially expressed in evg

The evergrowing phenotype of peach is caused by a major deletion affecting four of the six tandemly arranged *DAM* genes (Bielenberg et al., 2008). Our previous structural analysis of the *PavDAM* gene array in the sweet cherry cv ‘Cristobalina’, the wild-type parental cultivar of evg, revealed unique mutations when compared with other cultivars (Calle et al., 2021). Namely, specific SNPs in the coding sequences, a large deletion upstream of the *PavDAM1* gene, and various INDELS and SNPs in the UTR regions between *PavDAM4* and *PavDAM5*, that may be altering *PavDAMs* expression. These structural variants were shown to segregate with early blooming and are putatively associated with low chilling requirements and early flowering from this genotype (Calle et al., 2021). However, the precise cause of the low chilling phenotype of ‘Cristobalina’ remains to be elucidated. To confirm the structural conservation of the ‘Cristobalina’ *PavDAM* gene array in the wt progenies and the evg genotype, genomic DNA from all six *DAM* genes was amplified in these plant materials (**Supplementary Figure 2**). All six genes yielded the same expected PCR fragments of the gene body, in the tree types of materials (wild-type parental, wild-type seedlings, and evg cherry), indicating the absence of large structural mutations in the tandem array (**Supplementary Figure 2**). These results confirm that the evg cherry phenotype is not caused by the deletion of *DAM* genes as discovered in peach, and therefore the cause of the evg cherry phenotype is different from that of peach.

**Figure 3:**
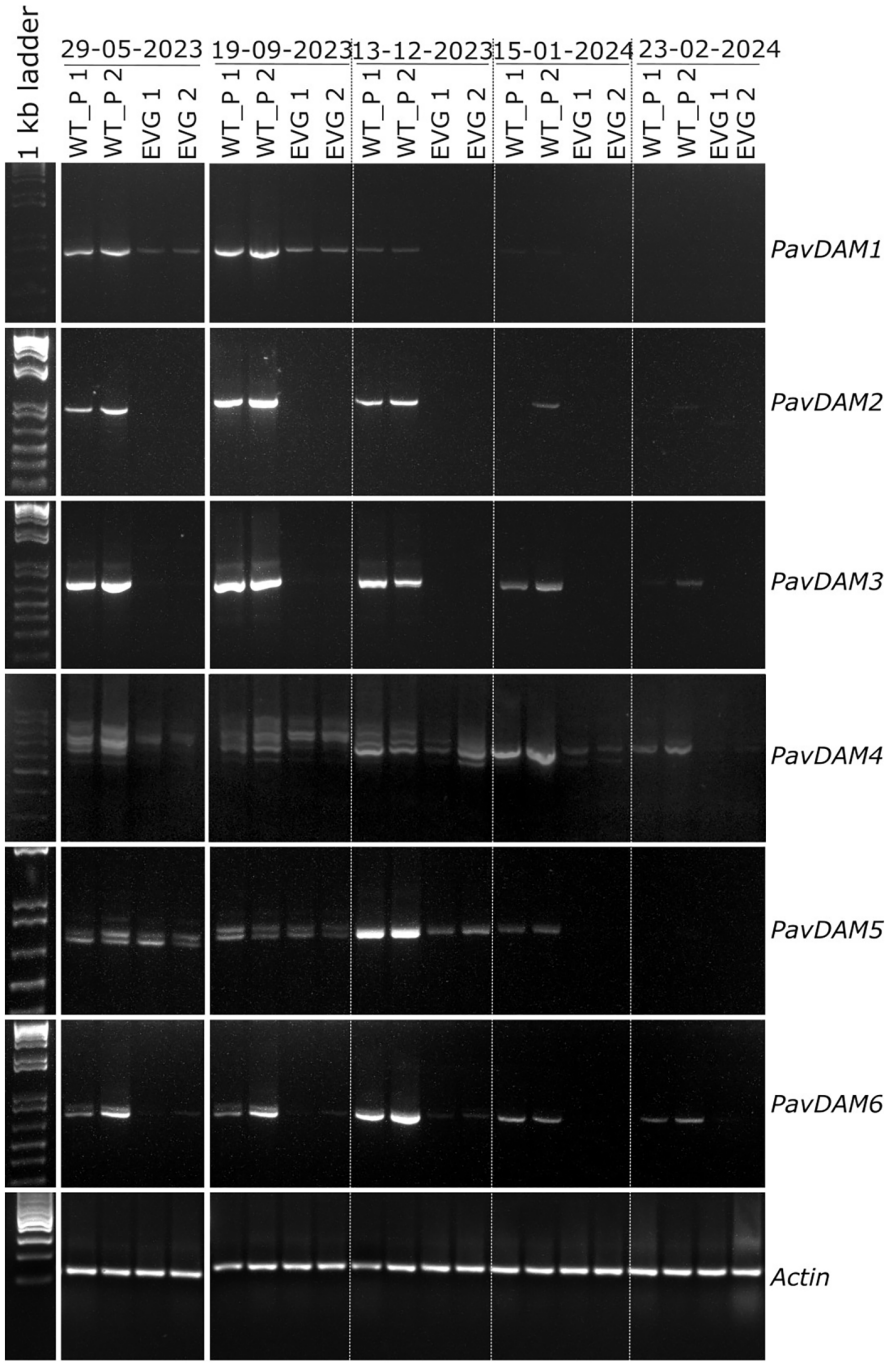
Time series RT-PCRs of *PavDAM* genes. RT-PCR was carried out for two wild type parental plants (wt-p1, wt-p2) and two evg clonal plants (evg-1, evg-2) in spring (May 2023) and throughout the 2023–2024 winter dormancy period (September to February). For better understanding, the individual images have been cropped and rearranged so that the time series is presented in chronological order (i.e., in the original images the May 2023 samples occupy the last lanes).

However, we cannot rule out that differential expression in *DAM* genes may be the cause of sweet cherry evg phenotype. Alterations in *DAM* genes underlie the evergrowing phenotype in other Rosaceous fruit trees (Bielenberg et al., 2008; Moser et al., 2020), and *DAMs* are generally considered to be the major integrators of winter dormancy in stone and pome fruits, so their expression could be affected in evg. To test this hypothesis, we performed RT-PCR gene expression analyses comparing the wild type (parental) and evg phenotype (**Figure 3**). Time-series RNA sampling of shoot apical meristem was carried in spring (May) and in the period between bud set in autumn (September) and bud burst the following spring (February). Consistent expression was observed for the reference gene *Actin* at the different genotypes, samples and times. However, differential expressions were observed between the evg and the wild type phenotypes for the *DAM* genes. Specifically, three distinct differential expression patterns could be distinguished. In the first pattern, affecting *DAM1, DAM5*, and *DAM6*, variable levels of down-regulation and time-limited expression was observed in the sweet cherry evg phenotype during the dormancy period. In the second pattern, involving *DAM2* and *DAM3*, a complete down-regulation is observed throughout the time series, including the spring sample, for the evg phenotype. In the third pattern, affecting *DAM4*, in addition to a slightly down-regulated expression observed in evg phenotype at some sampling times, differential alternative splicing appears to be at play with distinct differential patterns for both phenotypes. Thus, our results confirm the hypothesis of an putative impaired expression of *DAM* genes in the evg phenotype, but further research is needed to decipher which or to what extent these expression patterns are associated with or cause the evg phenotype.

Evergrowing phenotypes in Rosaceous woody plant species have been reported to be caused by a major deletion affecting four (*DAM1-4*) of the six *DAM* genes in peach (Bielenberg et al., 2008) and by RNAi-mediated down-regulation of *DAM1* in apple (Moser et al., 2020). It was shown that the *DAM1–4* genes consistently reach their maximum expression during bud set (Falavigna et al., 2019), and, thus, emerged as good candidates to explain the observed phenotype of absent bud set in evergrowing. But more generally, *DAM* genes have been shown to exhibit distinct seasonal expression patterns in several Rosaceous species (Falavigna et al., 2019), suggesting that different genes play roles in successive environmentally mediated seasonal growth regulation events, starting with bud set and growth cessation, then establishment and maintenance of endodormancy, and finally release from dormancy and bud break. Based on this assumption, *DAM5* and *DAM6*, which reach their maximum expression during winter dormancy (Falavigna et al., 2019), probably regulate dormancy maintenance and bud break (Falavigna et al., 2019; Lloret et al., 2021; Zhao et al., 2023). But the observation that overexpression of *DAM6* from Japanese apricot induces earlier growth cessation and bud set in poplar (Sasaki et al., 2011) and apple (Yamane et al., 2019) potentially challenges those assumptions. Thus, in addition to the known effects of *DAM1-4*, whether the sweet cherry evg phenotype is also associated with any of the patterns observed in *DAM5–6* warrants further research. Future studies should therefore focus on unravelling the function of each *DAM* gene, identifying their respective major interaction partners (e.g., if they interact with each other or with FLC or SVP), and ascertaining their regulatory networks.

### 3.4 Inbreeding depression as a cause of sweet cherry evg

Sweet cherry, like other Roseaceous species, is a highly heterozygous species. Heterozygosity is reinforced by a strict self-incompatibility system (gametopytic self-incompatibility) that prevents self- and cross-fertilization of genetically close individuals (reviewed in Herrero et al., 2017). Self-compatible mutants are scarce in this species, but the parental cultivar in this work is a local self-compatible landrace (Wünsch and Hormaza, 2004). The SNP genotyping of an F2 population (self-fertilization) from this cultivar, for the construction of a genetic map, revealed high levels of homozygosity, with large homozygous regions in the F2 population individuals, but also in the parental cultivar (Calle et al., 2018). Phenotypically, this population also exhibits symptoms of inbreeding depression like low vigor, early aging, and female and male sterility (Calle et al., 2022). In highly heterozygous species, high levels of homozygosity can lead to inbreeding depression due to overdominance, but mostly due to the expression of recessive deleterious alleles (Charlesworth and Charlesworth, 1999). The evg phenotype studied herein is a full-sib of this inbred population and its singular phenotype may be due to homozygosity in recessive dormancy-regulating alleles. Alternatively, inbreeding depression can also be driven by epigenetic changes (Vergeer et al., 2012). Furthermore, the low proportion of evergrowing phenotypes obtained after selfing (approx. 1/25) rules out the hypothesis of a single gene or locus (where 25% of the progeny should express the phenotype) and would rather point to a polygenic cause. Therefore, genes other than those in the *DAM*s cluster, both upstream and downstream of the dormancy regulating pathway, may be involved in the expression of the evg phenotype. To address this hypothesis, SNP and structural variant analyses involving evg versus a bulk of wild-type genotypes are warranted.

## 4 Conclusions

In summary, the sweet cherry evg phenotype reported here is the fourth natural mutant described in woody plant species (Thompson et al., 1985; Bielenberg et al., 2008; Harel-Beja et al., 2022). Although the precise molecular mechanism underlying this phenotype is still unknown, our preliminary analysis of *DAM* genes expression revealed expression patterns that -individually or in combination- may explain the observed phenotype. Importantly, this sweet cherry evergrowing phenotype is not due to a major deletion within the tandem set of *DAM* genes, as was observed in peach, making it valuable plant material to unravel *DAM* genes regulation of growth cessation and bud set, onsetting dormancy and bud break. Insight in this biological molecular mechanism(s) has great potential for understanding how woody plant species suspend their growth and development just at the right time to withstand the cold winter season before resuming growth with rising spring temperatures in temperate regions. This is especially important in the case of temperate Rosaceous fruit trees, where breeding for new varieties with low winter chill requirements became a major breeding goal for expanding cultivation areas and in the context of global warming.

## Data Availability

The original contributions presented in the study are included in the article/**Supplementary Material**. Further inquiries can be directed to the corresponding author.
