# Supplementary material for: An evergrowing sweet cherry for research and breeding
Source: Front Plant Sci. 2025 Nov 26;16:1677862. doi: 10.3389/fpls.2025.1677862 (PMC12689923; doi:10.3389/fpls.2025.1677862)
Supplement: Supplementary file 1 [file SupplementaryFile1.docx]

Supplementary Material

# Supplementary Figures and Tables

## Supplementary Figures


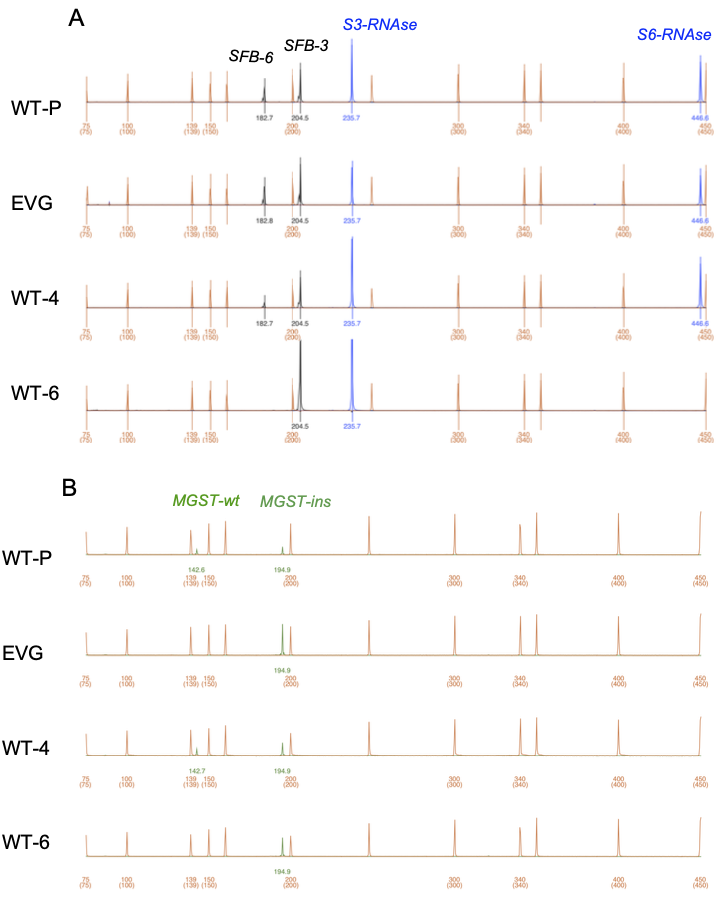


**Supplementary Figure 1.** Capillary electrophoresis (SeqStudio) of *S- RNase* and *SFB* alleles (A), and *MGST* alleles (B) in wild-type parental "Cristobalina" (wt-p), evergrowing cherry (evg), wild type sibling #4 and #6 (wt-4 and wt-6, respectively). Orange peaks correspond to size standard 500-LIZ, blue peaks correspond to 6-FAM labelled *S-RNase* fragment, black peaks correspond to NED labelled *SFB* fragment, green peaks correspond to VIC labelled *MGST* fragments. ‘-wt’: wild type allele; ‘-ins’: mutated allele with insertion conferring self-compatibility. Sizes in bp.


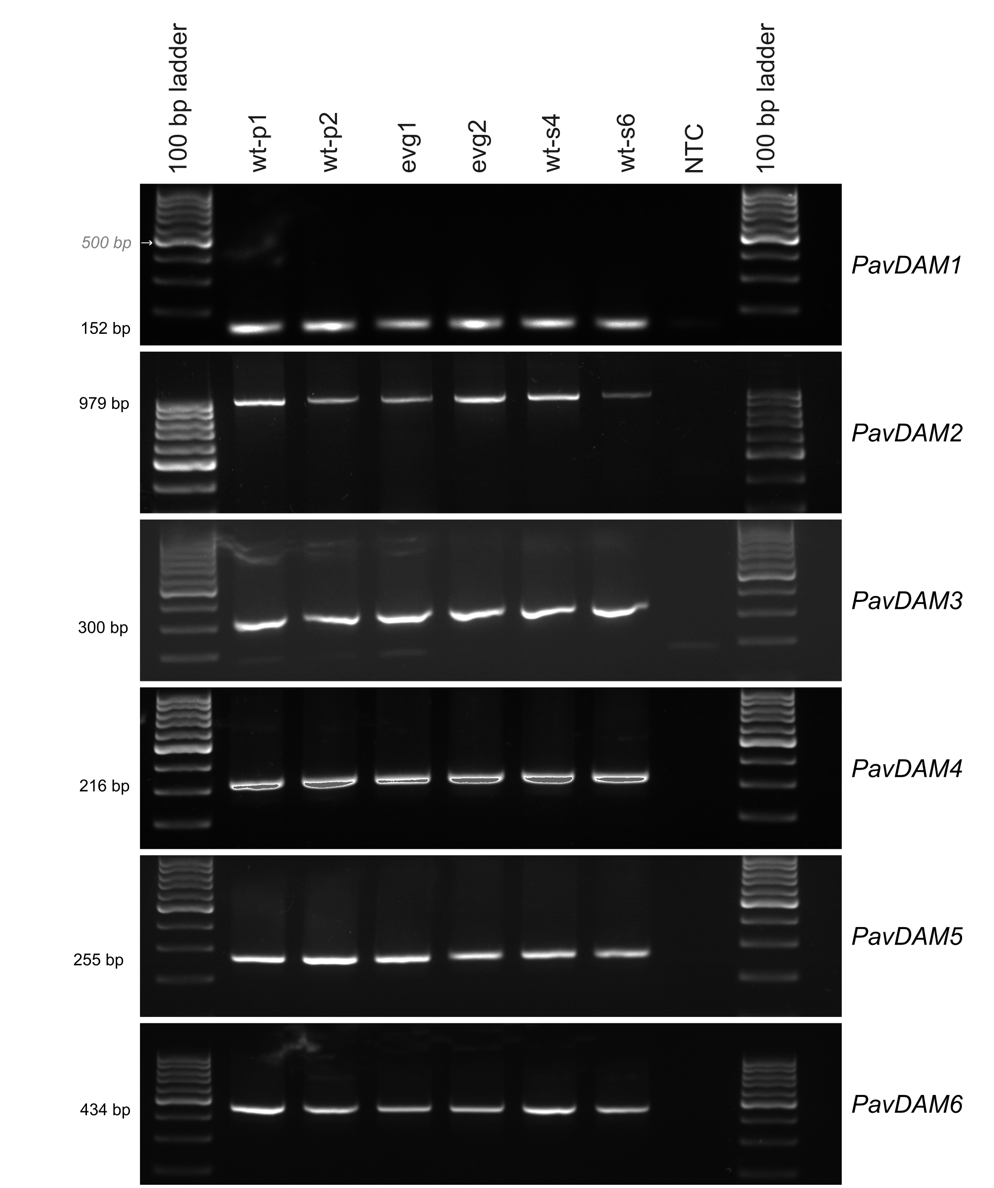


**Supplementary Figure 2**. No major *PavDAM* gene deletions are found in evg cherry. Amplification of genomic DNA of all 6 *PavDAM* genes in two wild type mother plants (wt-p1, wt-p2), two evergrowing clonal plants (evg1, evg2), and two wild type evg full sibs (wt-s4, wt-s6). Six primer combinations were used (see Table 1). Expected fragment sizes indicated on the left side of each gel. NTC: Non Template Control.

## Supplementary Tables

**Supplementary Table 1:** Primers sequences for *PavDAM* genomic DNA PCR amplification.

| Gene | Primer | Sequence (5’-3’) | Reference | Expected Size (bp) |
| --- | --- | --- | --- | --- |
| *PavDAM 1* | PavDAM1_qPCR_F | AGGGGACGATGAAAATGATGAGG | This work | 152 |
|  | PavDAM1_qPCR_R | GATAACTGCCACCTCAGATTCACA | This work |  |
| *PavDAM 2* | PavDAM2_qPCR_F | GTTAGAACAACTGGTGGACGCAAG | This work | 979 |
|  | PavDAM2_qPCR_R | AGCTGCTGATGGTTGCATTTGTGG | This work |  |
| *PavDAM 3* | PavDAM3_qPCR_F | AGCCAACAACCAGTTAAGGCAGAC | This work | 312 |
|  | PavDAM3_qPCR_R | GTCATCTCCAAGAGAGTGAGCACT | This work |  |
| *PavDAM 4* | PavDAM4_qPCR_F | AGCCAACAATCAGATGGTGATGTT | This work | 216 |
|  | PavDAM4_qPCR_R | CAMSAGAGAGACTGAGAGCACTGTT | This work |  |
| *PavDAM 5* | PavDAM5_qPCR_F | GAGAGGAAGAGTGCTCTTAATAAAAG | This work | 255 |
|  | PavDAM5_qPCR_R | GGGATAAAGTGACCTCATCGG | This work |  |
| *PavDAM 6* | PavDAM6_qPCR_F | GAGTGAGATCATGTCACTGGAGAA | This work | 434 |
|  | PavDAM6_qPCR_R | AGCTGGTAGAGGTGGCCATTGTG | This work |  |

**Supplementary Table 2:** Primers sequences for *PavDAMs* cDNA RT-PCR amplification.

| **Gene** | **Primer** | **Sequence (5’-3’)** | **Reference** | **Expected Size (bp)** |
| --- | --- | --- | --- | --- |
| ***PavDAM 1*** | PavDAM1_F_m | AGCAGCAGCAGCAGCCAAC | This work* | 815 |
|  | PavDAM1_R_m | CAATYACAACCCTCCACTT | This work* |  |
| ***PavDAM 2*** | PavDAM2_F | CATTTTAATCCCTTCCTTCG | Wang et al. 2020 | 1054 |
|  | PavDAM2_R_m | GGCTGCATGTAATACATAGGT | This work* |  |
| ***PavDAM 3*** | PavDAM3_ F | GGGATGGTGAAGATGATGAG | Wang et al. 2020 | 727 |
|  | PavDAM3_R1_m | CTTAACCAGAGAAATTAGGGAAGCC | This work* |  |
| ***PavDAM 4*** | PavDAM4_F_m | AACGAAGGGGGATGGTGAAAATGATG | This work* | 699 |
|  | PavDAM4_R_m | CTTAACCAAGGAAATTAKGGACGCC | This work* |  |
| ***PavDAM 5*** | PavDAM5_F_m | AACYCACCAAMGAAGATGATGAGG | This work* | 863 |
|  | PavDAM5_R_m | CTTTCTCCACTTCTTAACGCCASCA | This work* |  |
| ***PavDAM 6*** | PavDAM6_F_m | GACAAAGGGGAATGGTGAA | This work * | 753 |
|  | PavDAM6_R | TCCACTTCTTAACTAGGAAACTAGG | Wang et al. 2020 |  |
| ***Actin*** | ACT-F_Q1 | CGGTATTGCAGACGGATGAGC | Wang et al. 2013 | 133 |
|  | ACT-R-Q1 | GGTACTGAGGGATGCAAGGATGG | Wang et al. 2013 |  |

**Modified from Wang et al. 2020. A suffix _m (for modified) is added to original primer names.*

Wang, J., Zhang, X., Yan, G., Zhou, Y., and Zhang, K. (2013). Over-expression of the Q23

*PaAP1* gene from sweet cherry (Prunus avium L.) causes early flowering in Arabidopsis

thaliana. J. Plant Physiol. 170, 315–320. doi: 10.1016/j.jplph.2012.09.015
